# Bis(2-amino-3,5-di­chloro­pyridinium) hexa­chlorido­stannate(IV) dihydrate

**DOI:** 10.1107/S2414314622001912

**Published:** 2022-03-03

**Authors:** Rochdi Ghallab, Hassiba Bougueria, Hocine Merazig

**Affiliations:** a Environmental Molecular and Structural Chemistry Research Unit, University of Constantine-1, 25000, Constantine, Algeria; Benemérita Universidad Autónoma de Puebla, México

**Keywords:** crystal structure, hexa­chlorido­stannate(IV), pyridinium, X-ray diffraction

## Abstract

The title hybrid compound, (C_5_H_5_N_2_Cl_2_)_2_[SnCl_6_]·2H_2_O, was synthesized and its structure was identified by single-crystal X-ray diffraction. The structure is non-polymeric (0D) in terms of containing isolated [SnCl_6_]^2−^ polyhedra. The special position (0,0,0) of the Sn^IV^ atom in the crystal structure gives rise to a stacking structure with alternating cationic and anionic layers parallel to (001). The water mol­ecules are inter­calated between these layers, which are linked by cation–anion hydrogen bonds and dominant non-covalent inter­actions. The stability of the three-dimensional network for this compound is also discussed.

## Abstract 2

The crystal structure of a hybrid material containing 2-amino-3,5-di­chloro­pyridinium cations, a hexa­chlorido­stannate(IV) anion and water mol­ecules is described.

## Graphical abstract

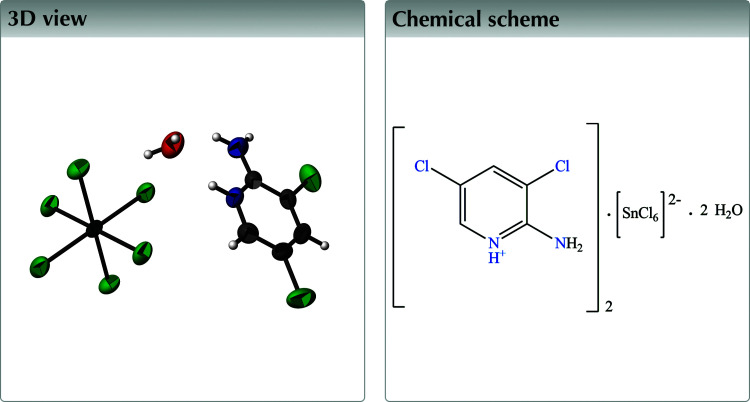

## Structure description

**Figure 1:**
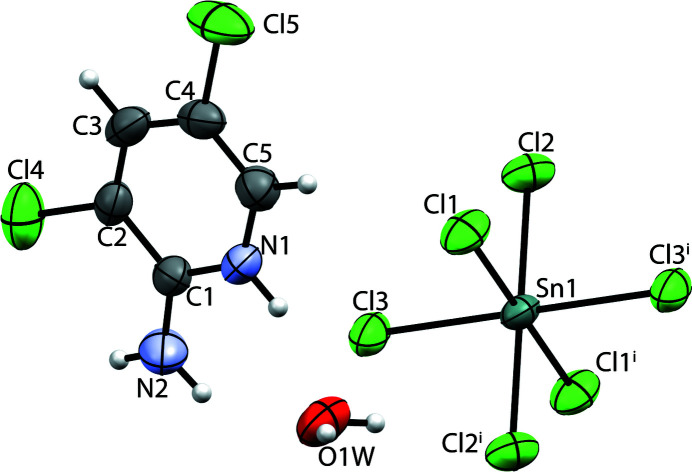
The molecular components in the crystal structure of the title compound, showing displacement ellipsoids at the 30% probability level [symmetry code: (i) −*x* + 2, −*y*, −*z*].

Bis(2-amino-3,5-di­chloro­pyridinium) hexa­chlorido­stannate(IV) dihydrate, (C_5_H_5_N_2_Cl_2_)_2_[SnCl_6_]·2H_2_O, crystallizes in the triclinic space group *P* 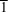 (Fig. 1 ▸). The tin(IV) atom is hexa­coordinated by chlorine atoms, generating a weakly distorted octa­hedron. The Sn—Cl bond lengths range from 2.4162 (5) to 2.4389 (5) Å while the Cl—Sn—Cl angles have a deviation of about ±1° [89.277 (19)–90.723 (19)°], see Table 1 ▸. These values are comparable to those of the same anion associated with other types of cations (Bouchene *et al.*, 2018 ▸). The absence of larger distortions can probably be attributed to the fact that the hexa­chlorido­stannate(IV) anions are free, *i.e.* none of the chloride ions are bridging, although they do accept N—H⋯Cl, O—H⋯Cl and C—H⋯Cl hydrogen bonds (Table 2 ▸).

**Figure 2:**
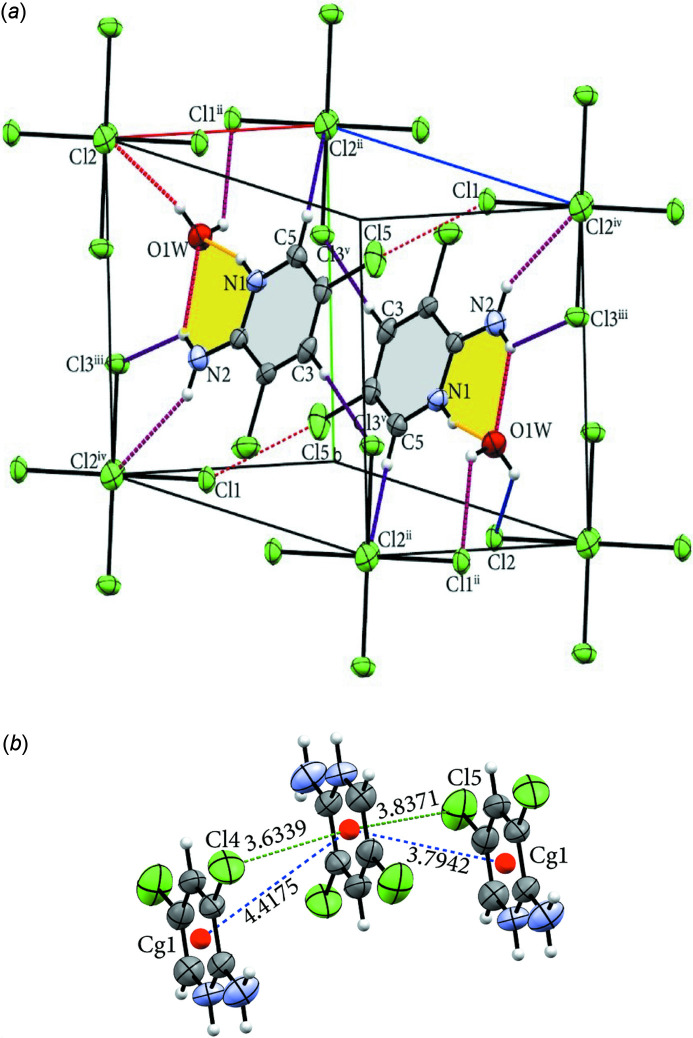
(*a*) Hydrogen bonds [yellow, purple and violet dashed lines; symmetry codes: (ii) −*x* + 1, −*y*, −*z*; (iii) *x*, *y* + 1, *z*; (iv) −*x* + 2, −*y* + 1, −*z*; (v) *x*, *y* + 1, *z* + 1] and halogen bonds (red dashed lines) in the title compound. (*b*) A view of the π-stacking inter­actions [blue dashed lines; symmetry codes: (i) 1 − *x*, 1 − *y*, 1 − *z*; (ii) 2 − *x*, 1 − *y*, 1 − *z*] and C—Cl⋯*Cg* [green dashed lines; symmetry operations: (i) 2 − *x*, 1 − *y*, 1 − *z*; (ii) 1 − *x*, 1 − *y*, 1 − *z*] inter­actions.

In the cation, we note an increase in C1—C2 and C2—Cl4 bond lengths and a decrease in C1—N2 bond lengths (Table 1 ▸). This phenomenon is due to resonance-assisted hydrogen bonding, commonly observed for this kind of mol­ecule (Bertolasi *et al.*, 1998 ▸). The C—N—C angle is 124.32 (17)°. This large angle can be attributed to the protonation of the N atom. These values are comparable with those of the same cation associated with other types of anions (Ghallab *et al.*, 2020 ▸). The inter­molecular inter­actions in the title compound were analysed using *PLATON* (Spek, 2020 ▸), which shows that the structural cohesion in the crystal structure is ensured by N—H⋯O, N—H⋯Cl, O—H⋯Cl and C—H⋯Cl hydrogen bonds (Fig. 2 ▸ *a*, Table 2 ▸). We also note the presence of Cl⋯Cl halogen bonds (Fig. 2 ▸ *a*), and of π-stacking inter­actions between centrosymmetrically related aromatic rings of the cations as well as Y—*X*⋯*Cg* inter­actions (Fig. 2 ▸ *b*).

## Synthesis and crystallization

Tin(II) chloride dihydrate (2.25 mmol) was mixed with 2-amino-3,5-di­chloro­pyridine (3.3 mmol) in 1:2 molar ratio and a few drops of hydro­chloric acid in an aliquot of distilled water were added. After stirring, the mixture was refluxed for one h at 343 K. After two weeks of slow solvent evaporation, single crystals suitable for X-ray analysis were obtained.

## Refinement

Crystal data, data collection and structure refinement details are summarized in Table 3 ▸.

## Supplementary Material

Crystal structure: contains datablock(s) I. DOI: 10.1107/S2414314622001912/bh4066sup1.cif

Structure factors: contains datablock(s) I. DOI: 10.1107/S2414314622001912/bh4066Isup2.hkl

CCDC reference: 2152891

Additional supporting information:  crystallographic information; 3D view; checkCIF report

## Figures and Tables

**Table 1:** Selected geometric parameters (Å, °)

Sn1—Cl1	2.4162 (5)	C1—N2	1.315 (3)
Sn1—Cl2	2.4389 (5)	C2—C3	1.356 (3)
Sn1—Cl3	2.4253 (5)	C2—Cl4	1.713 (2)
N1—C1	1.345 (3)	C3—C4	1.393 (3)
N1—C5	1.350 (3)	C4—C5	1.348 (3)
C1—C2	1.417 (3)	C4—Cl5	1.726 (2)
			
Cl1—Sn1—Cl2	90.722 (19)	N2—C1—N1	119.49 (18)
Cl1^i^—Sn1—Cl2	89.278 (19)	N2—C1—C2	124.50 (19)
Cl1—Sn1—Cl2^i^	89.277 (19)	C1—C2—Cl4	117.52 (16)
Cl1—Sn1—Cl3	89.906 (19)	C3—C2—C1	120.82 (18)
Cl1—Sn1—Cl3^i^	90.093 (19)	C3—C2—Cl4	121.66 (15)
Cl1^i^—Sn1—Cl3	90.093 (19)	C2—C3—C4	119.71 (18)
Cl3^i^—Sn1—Cl2	89.81 (2)	C3—C4—Cl5	120.22 (16)
Cl3—Sn1—Cl2	90.19 (2)	C5—C4—C3	119.70 (19)
Cl3—Sn1—Cl2^i^	89.81 (2)	C5—C4—Cl5	120.08 (18)
C1—N1—C5	124.32 (17)	C4—C5—N1	119.4 (2)
N1—C1—C2	116.00 (18)		

Symmetry code: (i) 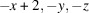 .

**Table 2:** Hydrogen-bond geometry (Å, °)

*D*—H⋯*A*	*D*—H	H⋯*A*	*D*⋯*A*	*D*—H⋯*A*
N1—H1⋯O1*W*	0.86	1.86	2.685 (2)	160
O1*W*—H1*WA*⋯Cl1^ii^	0.85	2.67	3.296 (2)	131
O1*W*—H1*WB*⋯Cl2	0.85	2.47	3.301 (2)	168
N2—H2*A*⋯Cl3^iii^	0.86	2.78	3.381 (2)	129
N2—H2*A*⋯O1*W*	0.86	2.38	3.065 (3)	137
N2—H2*B*⋯Cl2^iv^	0.86	2.67	3.435 (2)	149
C3—H3⋯Cl3^v^	0.93	2.77	3.695 (2)	177
C5—H5⋯Cl2^ii^	0.93	2.80	3.615 (2)	147

Symmetry codes: (ii) 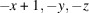 ; (iii) 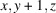 ; (iv) 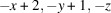 ; (v) 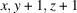 .

**Table 3:** Experimental details

Crystal data	
Chemical formula	(C_5_H_5_Cl_2_N_2_)_2_[SnCl_6_]·2H_2_O
*M*_r_	695.44
Crystal system, space group	Triclinic, *P* 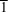
Temperature (K)	296
*a*, *b*, *c* (Å)	7.4624 (2), 8.4715 (2), 10.1324 (2)
α, β, γ (°)	101.434 (1), 90.043 (1), 107.554 (1)
*V* (Å^3^)	597.34 (2)
*Z*	1
Radiation type	Mo *K*α
μ (mm^−1^)	2.20
Crystal size (mm)	0.17 × 0.13 × 0.11

Data collection	
Diffractometer	Bruker APEXII CCD
Absorption correction	Multi-scan (*SADABS*; Bruker, 2016 ▸)
*T*_min_, *T*_max_	0.716, 0.785
No. of measured, independent and observed [*I* > 2σ(*I*)] reflections	13446, 3617, 3320
*R*_int_	0.017
(sin θ/λ)_max_ (Å^−1^)	0.714

Refinement	
*R*[*F* ^2^ > 2σ(*F* ^2^)], *wR*(*F* ^2^), *S*	0.025, 0.057, 1.02
No. of reflections	3617
No. of parameters	125
H-atom treatment	H-atom parameters constrained
Δρ_max_, Δρ_min_ (e Å^−3^)	0.43, −0.55

Computer programs: *APEX2* and *SAINT* (Bruker, 2016 ▸), *olex2.solve* (Bourhis *et al.*, 2015 ▸), *SHELXL* (Sheldrick, 2015 ▸) and *OLEX2* (Dolomanov *et al.*, 2009 ▸).

## full crystallographic data

### Crystal data

**Table:**

(C_5_H_5_Cl_2_N_2_)_2_[SnCl_6_]·2H_2_O	*Z* = 1
*M**_r_* = 695.44	*F*(000) = 338
Triclinic, *P*1	*D*_x_ = 1.933 Mg m^−^^3^
*a* = 7.4624 (2) Å	Mo *K*α radiation, λ = 0.71073 Å
*b* = 8.4715 (2) Å	Cell parameters from 8759 reflections
*c* = 10.1324 (2) Å	θ = 2.9–30.9°
α = 101.434 (1)°	µ = 2.20 mm^−^^1^
β = 90.043 (1)°	*T* = 296 K
γ = 107.554 (1)°	Block, clear light white
*V* = 597.34 (2) Å^3^	0.17 × 0.13 × 0.11 mm

### Data collection

**Table:**

Bruker APEXII CCD diffractometer	3320 reflections with *I* > 2σ(*I*)
φ and ω scans	*R*_int_ = 0.017
Absorption correction: multi-scan (SADABS; Bruker, 2016)	θ_max_ = 30.5°, θ_min_ = 3.6°
*T*_min_ = 0.716, *T*_max_ = 0.785	*h* = −10→10
13446 measured reflections	*k* = −11→12
3617 independent reflections	*l* = −14→14

### Refinement

**Table:**

Refinement on *F*^2^	Secondary atom site location: difference Fourier map
Least-squares matrix: full	Hydrogen site location: mixed
*R*[*F*^2^ > 2σ(*F*^2^)] = 0.025	H-atom parameters constrained
*wR*(*F*^2^) = 0.057	*w* = 1/[σ^2^(*F*_o_^2^) + (0.0221*P*)^2^ + 0.2999*P*] where *P* = (*F*_o_^2^ + 2*F*_c_^2^)/3
*S* = 1.02	(Δ/σ)_max_ < 0.001
3617 reflections	Δρ_max_ = 0.43 e Å^−^^3^
125 parameters	Δρ_min_ = −0.55 e Å^−^^3^
0 restraints	Extinction correction: SHELXL (Sheldrick 2015), Fc^*^=kFc[1+0.001xFc^2^λ^3^/sin(2θ)]^-1/4^
Primary atom site location: iterative	Extinction coefficient: 0.0164 (12)

### Special details

**Table:**

Refinement. Approximate positions for all H atoms were first obtained from difference Fourier maps. H atoms were then placed in idealized positions and refined using the riding-atom approximation: C—H = 0.93 Å and N—H = 0.86 Å, with *U*_iso_(H) = 1.2*U*_eq_(C,*N*). H atoms of the water molecule were located in a difference Fourier map and the water molecule geometry was eventually idealized, with O—H = 0.85 Å and *U*_iso_(H) = 1.5*U*_eq_(O).

### Fractional atomic coordinates and isotropic or equivalent isotropic displacement parameters (Å^2^)

**Table:**

	*x*	*y*	*z*	*U*_iso_*/*U*_eq_	
Sn1	1.000000	0.000000	0.000000	0.03672 (7)	
Cl1	0.75548 (7)	−0.01363 (7)	0.15744 (5)	0.05267 (13)	
Cl2	0.79968 (8)	0.04433 (7)	−0.17040 (5)	0.05363 (13)	
Cl3	0.87570 (9)	−0.30370 (6)	−0.08206 (5)	0.05831 (15)	
O1W	0.6070 (3)	0.3039 (2)	0.02149 (16)	0.0668 (5)	
H1WA	0.488075	0.275909	0.008232	0.100*	
H1WB	0.646535	0.225509	−0.021598	0.100*	
N1	0.6643 (3)	0.3678 (2)	0.29104 (16)	0.0475 (4)	
H1	0.636615	0.323122	0.206756	0.057*	
C1	0.7864 (3)	0.5252 (2)	0.32410 (19)	0.0423 (4)	
C2	0.8268 (3)	0.5946 (2)	0.4640 (2)	0.0440 (4)	
C3	0.7460 (3)	0.5045 (3)	0.55683 (19)	0.0500 (5)	
H3	0.773367	0.551264	0.648414	0.060*	
C4	0.6218 (3)	0.3417 (3)	0.5144 (2)	0.0485 (5)	
C5	0.5824 (3)	0.2753 (3)	0.3814 (2)	0.0504 (5)	
H5	0.499435	0.166763	0.352231	0.061*	
N2	0.8609 (3)	0.6033 (3)	0.2280 (2)	0.0635 (5)	
H2A	0.831417	0.553756	0.144754	0.076*	
H2B	0.939027	0.703933	0.248386	0.076*	
Cl4	0.97856 (10)	0.79620 (8)	0.51057 (8)	0.0752 (2)	
Cl5	0.51633 (12)	0.22629 (11)	0.63144 (8)	0.0814 (2)	

### Atomic displacement parameters (Å^2^)

**Table:**

	*U*^11^	*U*^22^	*U*^33^	*U*^12^	*U*^13^	*U*^23^
Sn1	0.04012 (10)	0.03823 (10)	0.02500 (8)	0.00279 (7)	0.00185 (6)	0.00539 (6)
Cl1	0.0505 (3)	0.0630 (3)	0.0385 (2)	0.0095 (2)	0.0146 (2)	0.0090 (2)
Cl2	0.0537 (3)	0.0674 (3)	0.0377 (2)	0.0142 (2)	−0.0065 (2)	0.0131 (2)
Cl3	0.0850 (4)	0.0373 (2)	0.0378 (2)	0.0008 (2)	0.0014 (2)	0.00182 (18)
O1W	0.0687 (10)	0.0721 (11)	0.0450 (8)	0.0080 (8)	−0.0052 (7)	0.0001 (8)
N1	0.0607 (10)	0.0453 (9)	0.0327 (7)	0.0142 (8)	0.0013 (7)	0.0026 (6)
C1	0.0504 (10)	0.0414 (9)	0.0383 (9)	0.0185 (8)	0.0079 (8)	0.0083 (7)
C2	0.0461 (10)	0.0422 (9)	0.0421 (9)	0.0178 (8)	−0.0021 (8)	−0.0014 (8)
C3	0.0571 (12)	0.0667 (13)	0.0328 (8)	0.0331 (10)	−0.0002 (8)	0.0038 (8)
C4	0.0546 (12)	0.0595 (12)	0.0439 (10)	0.0288 (10)	0.0119 (9)	0.0216 (9)
C5	0.0538 (12)	0.0443 (10)	0.0524 (11)	0.0132 (9)	0.0055 (9)	0.0112 (9)
N2	0.0852 (14)	0.0542 (11)	0.0511 (10)	0.0171 (10)	0.0201 (10)	0.0175 (9)
Cl4	0.0688 (4)	0.0496 (3)	0.0897 (5)	0.0096 (3)	−0.0104 (3)	−0.0128 (3)
Cl5	0.0971 (5)	0.1009 (5)	0.0742 (4)	0.0474 (4)	0.0359 (4)	0.0562 (4)

### Geometric parameters (Å, º)

**Table:**

Sn1—Cl1	2.4162 (5)	C1—C2	1.417 (3)
Sn1—Cl1^i^	2.4162 (5)	C1—N2	1.315 (3)
Sn1—Cl2	2.4389 (5)	C2—C3	1.356 (3)
Sn1—Cl2^i^	2.4389 (5)	C2—Cl4	1.713 (2)
Sn1—Cl3	2.4253 (5)	C3—H3	0.9300
Sn1—Cl3^i^	2.4253 (5)	C3—C4	1.393 (3)
O1W—H1WA	0.8499	C4—C5	1.348 (3)
O1W—H1WB	0.8496	C4—Cl5	1.726 (2)
N1—H1	0.8600	C5—H5	0.9300
N1—C1	1.345 (3)	N2—H2A	0.8600
N1—C5	1.350 (3)	N2—H2B	0.8600
			
Cl1—Sn1—Cl1^i^	180.0	N1—C1—C2	116.00 (18)
Cl1—Sn1—Cl2	90.722 (19)	N2—C1—N1	119.49 (18)
Cl1^i^—Sn1—Cl2	89.278 (19)	N2—C1—C2	124.50 (19)
Cl1—Sn1—Cl2^i^	89.277 (19)	C1—C2—Cl4	117.52 (16)
Cl1^i^—Sn1—Cl2^i^	90.723 (19)	C3—C2—C1	120.82 (18)
Cl1—Sn1—Cl3	89.906 (19)	C3—C2—Cl4	121.66 (15)
Cl1—Sn1—Cl3^i^	90.093 (19)	C2—C3—H3	120.1
Cl1^i^—Sn1—Cl3^i^	89.907 (19)	C2—C3—C4	119.71 (18)
Cl1^i^—Sn1—Cl3	90.093 (19)	C4—C3—H3	120.1
Cl2^i^—Sn1—Cl2	180.0	C3—C4—Cl5	120.22 (16)
Cl3^i^—Sn1—Cl2	89.81 (2)	C5—C4—C3	119.70 (19)
Cl3^i^—Sn1—Cl2^i^	90.19 (2)	C5—C4—Cl5	120.08 (18)
Cl3—Sn1—Cl2	90.19 (2)	N1—C5—H5	120.3
Cl3—Sn1—Cl2^i^	89.81 (2)	C4—C5—N1	119.4 (2)
Cl3—Sn1—Cl3^i^	180.0	C4—C5—H5	120.3
H1WA—O1W—H1WB	109.5	C1—N2—H2A	120.0
C1—N1—H1	117.8	C1—N2—H2B	120.0
C1—N1—C5	124.32 (17)	H2A—N2—H2B	120.0
C5—N1—H1	117.8		
			
N1—C1—C2—C3	0.4 (3)	C5—N1—C1—C2	−0.6 (3)
N1—C1—C2—Cl4	−178.93 (15)	C5—N1—C1—N2	178.8 (2)
C1—N1—C5—C4	0.3 (3)	N2—C1—C2—C3	−179.0 (2)
C1—C2—C3—C4	0.0 (3)	N2—C1—C2—Cl4	1.7 (3)
C2—C3—C4—C5	−0.3 (3)	Cl4—C2—C3—C4	179.31 (16)
C2—C3—C4—Cl5	−179.42 (16)	Cl5—C4—C5—N1	179.25 (16)
C3—C4—C5—N1	0.1 (3)		

Symmetry code: (i) −*x*+2, −*y*, −*z*.

### Hydrogen-bond geometry (Å, º)

**Table:**

*D*—H···*A*	*D*—H	H···*A*	*D*···*A*	*D*—H···*A*
N1—H1···O1*W*	0.86	1.86	2.685 (2)	160
O1*W*—H1*WA*···Cl1^ii^	0.85	2.67	3.296 (2)	131
O1*W*—H1*WB*···Cl2	0.85	2.47	3.301 (2)	168
N2—H2*A*···Cl3^iii^	0.86	2.78	3.381 (2)	129
N2—H2*A*···O1*W*	0.86	2.38	3.065 (3)	137
N2—H2*B*···Cl4	0.86	2.61	2.986 (2)	108
N2—H2*B*···Cl2^iv^	0.86	2.67	3.435 (2)	149
C3—H3···Cl3^v^	0.93	2.77	3.695 (2)	177
C5—H5···Cl2^ii^	0.93	2.80	3.615 (2)	147

Symmetry codes: (ii) −*x*+1, −*y*, −*z*; (iii) *x*, *y*+1, *z*; (iv) −*x*+2, −*y*+1, −*z*; (v) *x*, *y*+1, *z*+1.
